# Supplementary material for: CRISPRi with barcoded expression reporters dissects regulatory networks in human cells
Source: bioRxiv. 2024 Sep 6:2024.09.06.611573. Preprint. [Version 1] doi: 10.1101/2024.09.06.611573 (PMC11398470; doi:10.1101/2024.09.06.611573)
Supplement: Supplement 2 [file NIHPP2024.09.06.611573v1-supplement-2.pdf]

bioRxiv preprint doi: https://doi.org/10.1101/2024.09.06.611573; this version posted September 6, 2024. The copyright holder for this preprint (which was not certified by peer review) is the author/funder, who has granted bioRxiv a license to display the preprint in perpetuity. It is made available under aCC-BY 4.0 International license.

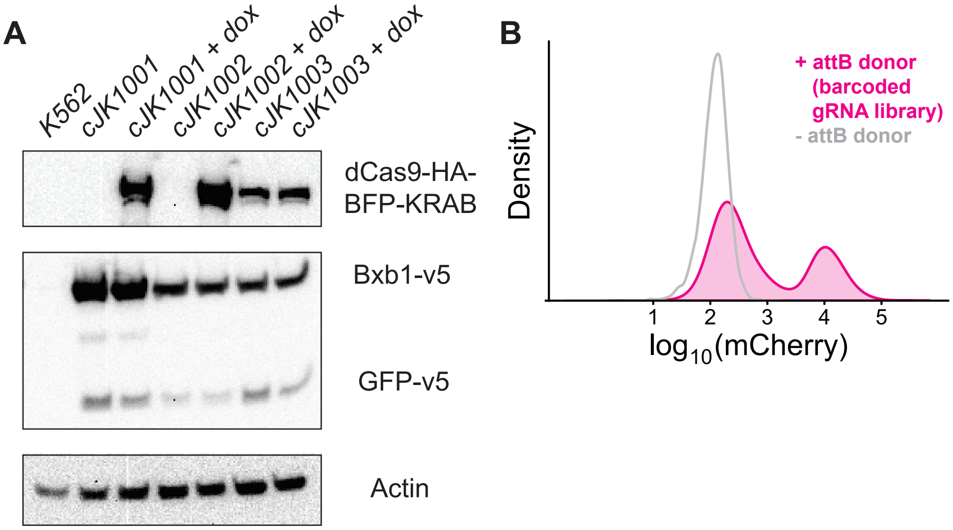

Supplementary Figure 1: Further validation of mammalian CiBER-seq components. (A) Western Blot showing either inducible or constitutive dCas9-KRAB expression, Bxb1 or GFP expression, and actin as loading control. Cells were either left untreated or treated with 1 μg/mL doxycycline for 72 hrs. cJK1001: K562 cells with inducible dCas9-KRAB, two copies of attP landing pad. cJK1002: K562 cells with inducible dCas9-KRAB, one copy of attP landing pad. cJK1003: K562 cells with constitutive dCas9-KRAB, one copy of attP landing pad. **(B)** Cells that integrate the barcoded sgRNA library also express mCherry.

bioRxiv preprint doi: https://doi.org/10.1101/2024.09.06.611573; this version posted September 6, 2024. The copyright holder for this preprint (which was not certified by peer review) is the author/funder, who has granted bioRxiv a license to display the preprint in perpetuity. It is made available under aCC-BY 4.0 International license.

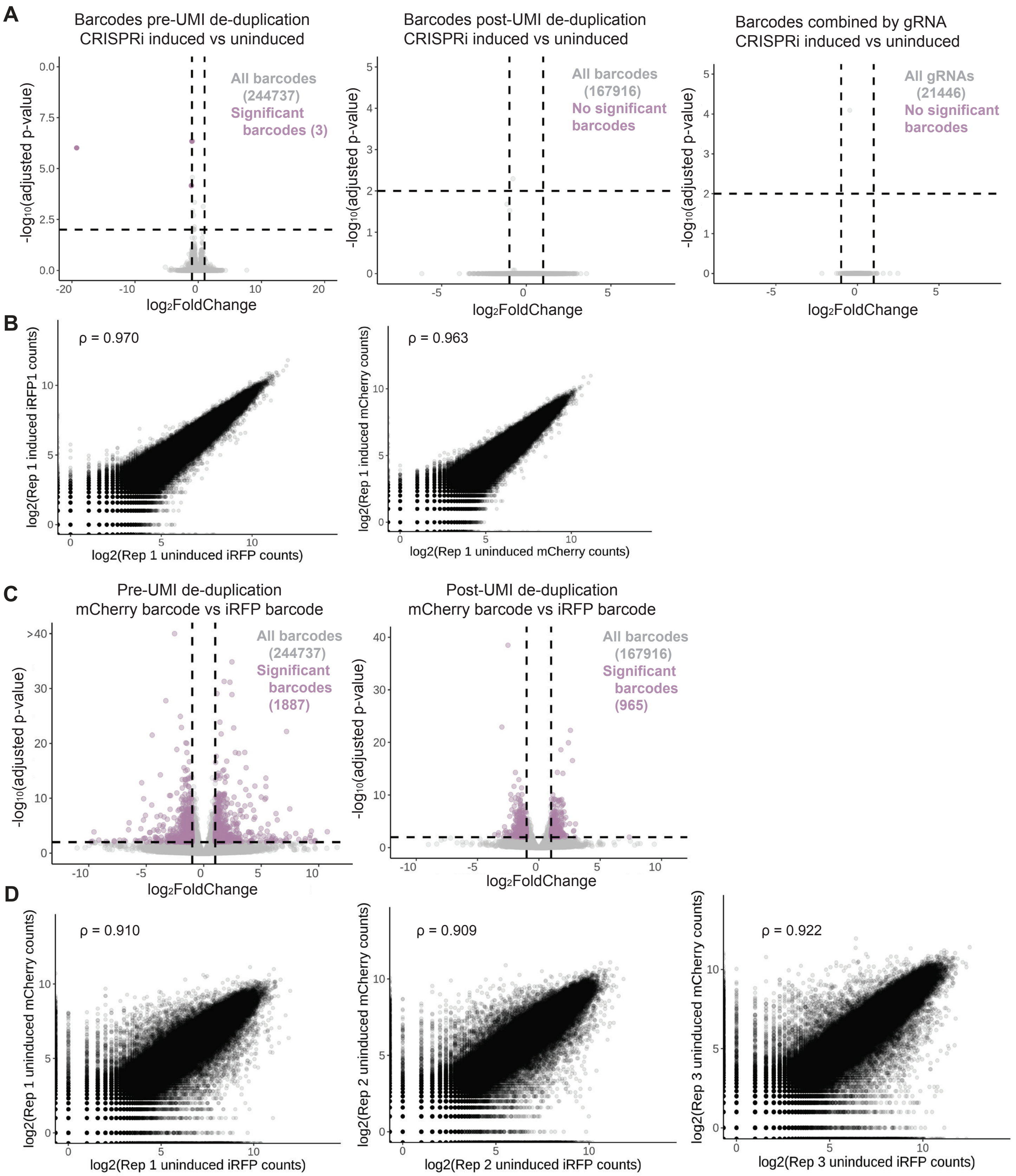

bioRxiv preprint doi: https://doi.org/10.1101/2024.09.06.611573; this version posted September 6, 2024. The copyright holder for this preprint (which was not certified by peer review) is the author/funder, who has granted bioRxiv a license to display the preprint in perpetuity. It is made available under aCC-BY 4.0 International license.

Supplementary Figure 2: Additional validation of CiBER-seq screen for matched barcoded reporters. (A) DESeq2 comparison of CRISPRi induced vs uninduced samples for barcodes pre-UMI de-duplication, post-UMI de-duplication, and post combining barcodes by sgRNA. For the first two panels, each point represents one barcode, and for the last panel, each point represents one sgRNA. Dashed lines represent adjusted p-value < 0.01 and log2-fold change > 1 or < -1. **(B)** Comparison of either iRFP or mCherry barcode counts between uninduced and induced CRISPRi samples for replicate 1. Each point represents one barcode. **(C)** DESeq2 comparison of mCherry vs iRFP changes for barcodes pre-UMI de-duplication and post-UMI de-duplication (from both the pre- and post-CRISPRi induction samples). Each point represents one barcode. Dashed lines represent adjusted p-value < 0.01 and log2-fold change > 1 or < -1. **(D)** Comparison of iRFP and mCherry barcode counts for each replicate. Each point represents one barcode.

bioRxiv preprint doi: https://doi.org/10.1101/2024.09.06.611573; this version posted September 6, 2024. The copyright holder for this preprint (which was not certified by peer review) is the author/funder, who has granted bioRxiv a license to display the preprint in perpetuity. It is made available under aCC-BY 4.0 International license.

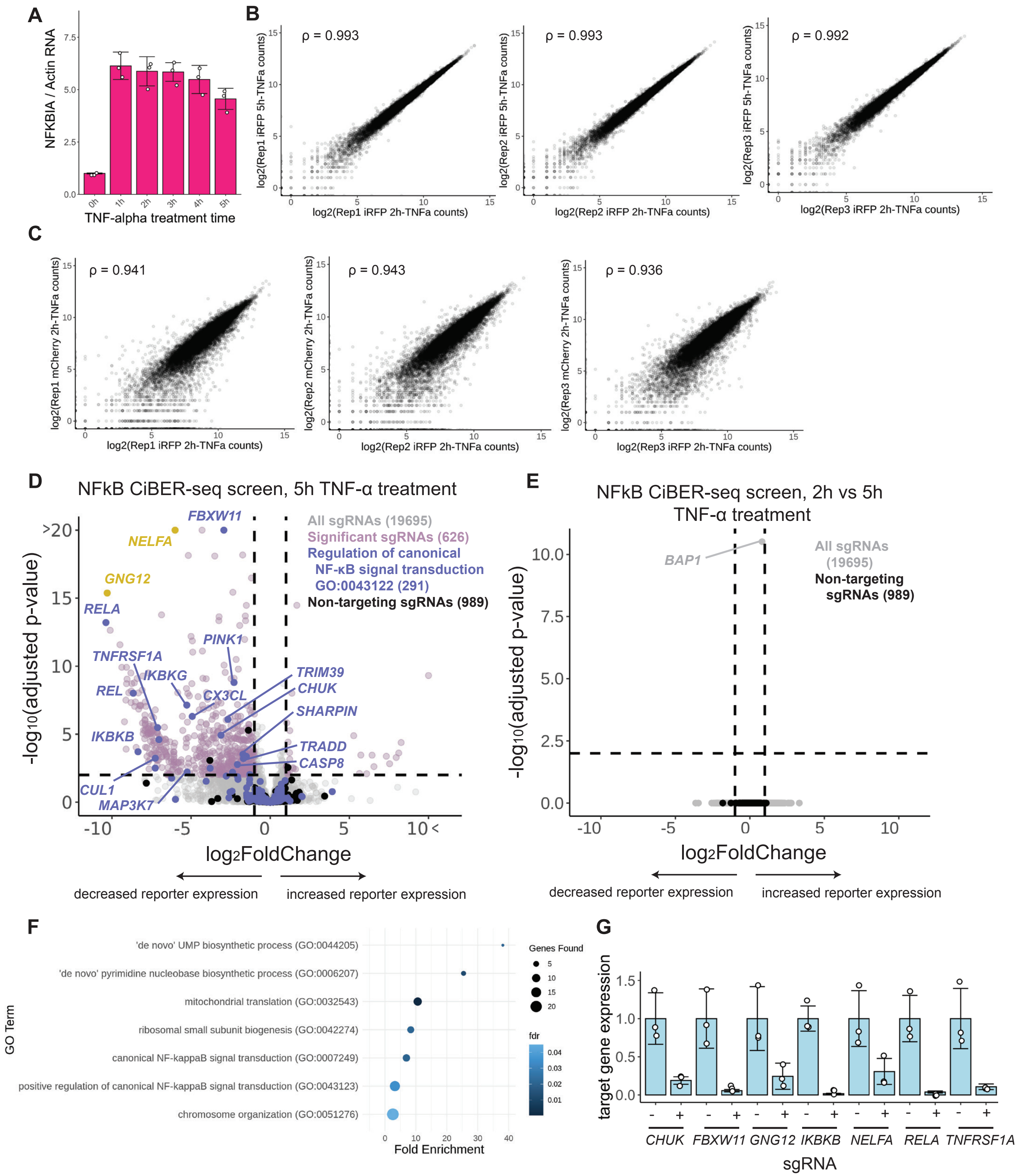

bioRxiv preprint doi: https://doi.org/10.1101/2024.09.06.611573; this version posted September 6, 2024. The copyright holder for this preprint (which was not certified by peer review) is the author/funder, who has granted bioRxiv a license to display the preprint in perpetuity. It is made available under aCC-BY 4.0 International license.

Supplementary Figure 3: Additional validation of NF-κB CiBER-seq screen. (A) RT-qPCR of *NFKBIA* normalized to *ACTB* across different timepoints of TNF-alpha stimulation (n=3). **(B)** Comparison of iRFP barcode counts between the 2-hr and 5-hr timepoints. Each point represents an sgRNA. **(C)** Comparison of iRFP and mCherry barcode counts. Each point represents an sgRNA. **(D)** DESeq2 analysis as in Fig 3E. **(E)** DESeq2 analysis of genome-wide CiBER-seq screen for regulators of NF-κB, comparing the 2-hr and 5-hr timepoints. Each point represents a single sgRNA. Dashed lines represent adjusted p-value < 0.01 and log2-fold change > 1 or < -1. **(F)** Gene ontology statistical overrepresentation analysis of guides that prevent mCherry induction after 2 hrs of TNF-α treatment (log2FoldChange < -1 and adjusted p-value < 0.01). **(G)** RT-qPCR of each indicated endogenous gene normalized to *ACTB* in cells with and without the indicated, corresponding sgRNA (n=3).
